# Biomechanical differences between females and males with patellofemoral pain: A systematic review and meta‐analysis

**DOI:** 10.1002/jeo2.70880

**Published:** 2026-08-21

**Authors:** Lucas Pallone, Carolline P. Nunes, Felipe F. Gonzalez, Talissa O. Generoso, Rodrigo Saad‐Berreta, Kaitlyn M. Joyce, Eliane C. Guadagnin, Jorge Chahla, Jonathan A. Gustafson, Leonardo Metsavaht, Gustavo Leporace

**Affiliations:** ^1^ Department of Sports Medicine Midwest Orthopaedics at Rush University Medical Center Chicago Illinois USA; ^2^ Deparment of Biomechanics research Instituto Brasil de Tecnologias da Saúde Rio de Janeiro RJ Brazil; ^3^ Departamento de Ortopedia e Traumatologia Universidade de São Paulo Ribeirão Preto SP Brazil; ^4^ Departamento de Diagnóstico por Imagem Universidade Federal de São Paulo São Paulo SP Brazil; ^5^ Department of Orthopedic Surgery Rush University Medical Center Chicago Illinois USA

**Keywords:** biomechanics, gender differences, kinematics, kinetics, patellofemoral pain

## Abstract

**Purpose:**

Patellofemoral pain (PFP) is more prevalent in females and altered lower limb biomechanics have been implicated in its development and persistence. However, it remains unclear whether movement patterns differ between men and women with PFP during functional activities. Many biomechanical studies rely on mixed‐sex cohorts, potentially obscuring sex‐specific movement patterns. Therefore, this study aimed to systematically review biomechanical differences between females and males with PFP during functional tasks.

**Methods:**

PubMed/MEDLINE, Embase, Scopus, CINAHL, Cochrane CENTRAL, Cochrane Database of Systematic Reviews and Google Scholar were searched from inception to 10 September 2025. Studies comparing in vivo biomechanical characteristics of females and males with PFP during functional tasks were included, whereas studies using mixed‐sex cohorts without sex‐stratified analysis or focusing exclusively on a single sex were excluded. Extracted data comprised participant demographics, methodological characteristics and kinetic and kinematic outcomes. Methodological quality was assessed using a modified Downs and Black checklist. Random‐effects meta‐analyses were performed using standardized mean differences.

**Results:**

Five cross‐sectional studies including 170 individuals with PFP (84 females and 86 males) were analysed. Tasks evaluated included single‐leg squat, step‐down, mini‐squat and single‐leg jumping activities. Meta‐analysis of single‐leg squat data showed greater peak hip adduction in females compared with males (mean difference 4.93°, 95% confidence interval [CI] 2.60–7.25; *I* ^2^ = 0%). Females also demonstrated greater peak knee abduction (mean difference 5.74°, 95% CI 2.31–9.16; *I* ^2^ = 66%). No significant differences were observed for contralateral pelvic drop. Qualitative synthesis indicated greater frontal‐plane kinematics in females, whereas males demonstrated greater hip, knee and ankle extension moments during jumping tasks. Several findings were based on single studies.

**Conclusions:**

Only a limited number of studies evaluated sex‐specific biomechanics in individuals with PFP. Despite the small number of studies, consistent differences between females and males were observed, suggesting that sex influences movement strategies and should be considered in biomechanical assessment and rehabilitation.

**Level of Evidence:**

Level IV.

**Abbreviations**
BMI: body mass index
CI: confidence interval
HQ: high quality
MQ: moderate quality
PFP: patellofemoral pain
PRISMA: Preferred Reporting Items for Systematic Reviews and Meta‐Analyses
SD: standard deviation
SMD: standardized mean differences

## INTRODUCTION

Patellofemoral pain (PFP) is a prevalent and often persistent musculoskeletal condition [7, 19] that affects approximately 23% of the adult population [37], imposing a considerable social and economic burden. Individuals with PFP frequently report reduced levels of physical activity and declines in quality of life and overall health [6, 18, 36]. Epidemiological evidence further indicates that PFP is more prevalent in females, who are reported to be approximately twice as likely to experience PFP compared with males [37]. This consistent sex‐related disparity has raised interest in whether differences between females and males may contribute to variations in patellofemoral joint loading and symptom development.

The aetiology of PFP is multifactorial, encompassing anatomical, biomechanical, psychological, social and behavioural factors [11, 34, 40, 45]. Although the interactions among these factors and the clinical manifestation of PFP remain incompletely understood, growing evidence consistently links altered lower‐limb biomechanics to PFP, suggesting that biomechanical impairments may play a pivotal role in both symptom development and persistence [4, 13, 14, 15, 16, 20, 33, 38]. In parallel, studies in healthy populations have demonstrated sex‐related differences in movement strategies, with females often exhibiting greater hip adduction, femoral internal rotation and dynamic knee valgus during weight‐bearing activities [17, 22, 32, 35, 39, 46]. These findings suggest that biomechanical patterns may differ between females and males and could contribute to the observed differences in PFP prevalence.

Despite this evidence, many biomechanical studies in PFP continue to rely on mixed‐sex cohorts, potentially obscuring sex‐specific movement characteristics [1, 5]. When sexes are evaluated separately, studies most commonly include only female participants, without comparison with a male PFP group [9, 23, 43]. As a result, direct comparisons between females and males with PFP remain limited. Clarifying biomechanical distinctions between females and males is therefore essential to better understand their potential contribution to the higher incidence of PFP in females. Such insights are critical for informing the development of sex‐specific prevention and rehabilitation strategies, as well as for establishing parameters to guide future biomechanical research.

Therefore, this systematic review and meta‐analysis aims to synthesize the current evidence regarding biomechanical differences between males and females with PFP during functional activities.

## METHODS

### Manuscript registration

This systematic review was conducted in accordance with the Preferred Reporting Items for Systematic Reviews and Meta‐Analyses (PRISMA 2020) guidelines (Supporting Information S1: Material 1) [21]. The review protocol was registered in the International Prospective Register of Systematic Reviews (PROSPERO) under the registration number CRD42024518401.

### Eligibility criteria

This systematic review included studies that assessed the biomechanics of both males and females with insidious onset of PFP symptoms, unrelated to a traumatic event. Studies were included if they met the following criteria: (a) reported kinetic or kinematic data from functional tasks, such as single‐leg or double‐leg squats, stair ascent or descent, step‐down, single‐leg or double‐leg jumps, lunging or drop jumps; (b) reported biomechanical outcomes separately by sex; (c) cross‐sectional design, longitudinal studies and clinical trials reporting baseline biomechanical assessments before any intervention were also included. Studies with mixed‐sex cohorts not reporting outcomes separately by sex or studying only one sex without comparison, and those focusing on patients with patellofemoral osteoarthritis or patellar instability were excluded. Additionally, studies involving patients who underwent any surgical treatment before the biomechanical assessment, such as total or partial knee replacements, were not considered.

### Search methods and study selection

A comprehensive literature search was developed by the authors and was run by an experienced medical librarian (J.C.W.) from inception to 10 September 2025, in the following databases: PubMed/MEDLINE, Embase, Scopus, CINAHL, Cochrane CENTRAL Register of Controlled Trials and the Cochrane Database of Systematic Reviews, in addition to Google Scholar (Table 1, Supporting Information S1: Material 2). An additional search of ClinicalTrials.gov (U.S. National Library of Medicine) was performed on 3 April 2026 following peer‐review comments. Both controlled vocabularies (e.g., MeSH terms) and keywords in the title or abstract fields were searched. There were no restrictions on geography, sex or age of participants or language of publication. Additionally, a hand search was conducted of the reference lists of selected articles.

**Table 1:** Search strategy used for the identification of studies investigating biomechanical differences between females and males with patellofemoral pain during functional tasks.

Patellofemoral joint	Disorder	Task	Biomechanics
Patellofemoral	Pain	Squat	Biomechanics
Patello‐femoral	Syndrome	Lunge	Kinematic
Retropatellar	Chondromalacia	Stair	Kinetic
Femoropatellar	Dysfunction	Step	
Femoro‐patellar		Jump	
Anterior knee			

*Note*: Terms within each column were combined using the Boolean operator ‘OR’, while terms across columns were combined using ‘AND’. The search strategy was adapted according to the indexing system and search requirements of each database.

The search results were exported to a reference management database, and two independent reviewers (L.P. and C.P.N.) screened all titles and abstracts to identify studies for full‐text assessment. Each reviewer assessed the entire set of retrieved records. Studies selected by at least one reviewer were retrieved for full‐text review. Full‐text eligibility assessment was conducted independently by the same reviewers (L.P. and C.P.N.) according to the predefined inclusion criteria. Disagreements were resolved by a third reviewer (F.F.G.). Studies that met all eligibility criteria were included in the systematic review.

### Data extraction

Data extraction was performed independently by two reviewers (L.P. and C.P.N.). A third reviewer (F.F.G.) checked the extracted data and resolved any discrepancies. Characteristics of the participants, equipment used, data collection methods and kinetic and kinematic outcomes from functional tasks were extracted using a standardized form. For studies that included additional groups (e.g., healthy participants), only data from participants with PFP were extracted.

### Methodological quality and risk of bias in individual studies assessment

Methodological quality was assessed by two independent reviewers using a 15‐item modified Downs and Black checklist [12], previously validated in similar reviews [2, 8, 26, 29]. Disagreements were resolved by consensus. Each question received a rating of 1 for a ‘yes’ response and 0 for a ‘no’ or ‘unable to assess’ response, except for Question 4, which was assigned a score of 2 for a ‘yes’ response, 1 for a ‘partial’ response and 0 for a ‘no’ response. Studies were categorized as high (≥11), moderate (6–10) or low quality (≤5).

### Risk of bias of the systematic review

Risk of bias was independently assessed by two reviewers using the ROBIS (Risk Of Bias In Systematic Reviews) tool, with disagreements resolved by consensus. ROBIS evaluates potential bias in the review process across four domains: study eligibility criteria, identification and selection of studies, data collection and study appraisal and synthesis and findings. Each domain was classified as presenting low, high or unclear concern for bias according to ROBIS guidance, followed by an overall judgement of risk of bias for the review.

### Statistical analysis

Meta‐analyses were performed only when studies reported comparable biomechanical outcomes using similar functional tasks and measurement methods. Studies evaluating different functional tasks were synthesized qualitatively and were not pooled in the same meta‐analysis. Pooled statistics were computed using inverse‐variance weighting, assuming a random‐effects model. Standardized mean differences (SMDs) in joint kinematics between female and male groups were the primary measure of effect size.

Heterogeneity was quantified using the DerSimonian–Laird method (*τ* ^2^) [10] and *I* ^2^, and *χ* ^2^ test in accordance with the guidelines delineated in the Cochrane manual [30]. The *χ* ^2^ test was interpreted as statistically significant if *p* < 0.05. *I* ^2^ statistics followed the Cochrane manual for interpretation: 0%–40% might not be important; 30%–60% may represent moderate heterogeneity; 50%–90% may represent substantial heterogeneity; 75%–100%, considerable heterogeneity [10, 25]. *p* value and 95% confidence intervals (CIs) were calculated. *p* value was considered statistically significant when *p* < 0.05.

All data analysis was performed using R software (Version 4.4.3; R Foundation for Statistical Analysis). Variables not eligible for quantitative synthesis were analysed descriptively.

## RESULTS

### Study selection

**Figure 1:**
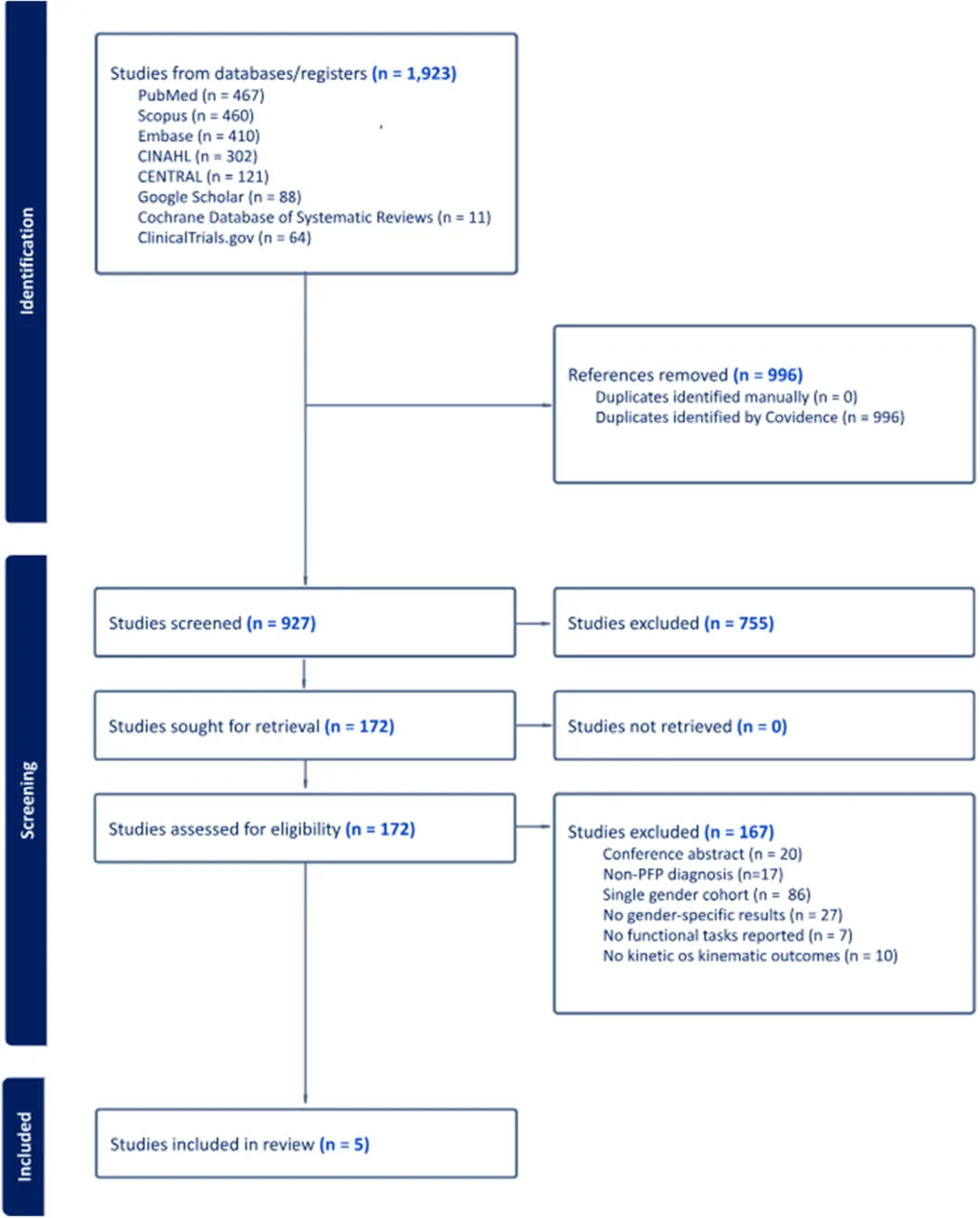
PRISMA flow diagram illustrating the study selection process for the systematic review and meta‐analysis investigating biomechanical differences between females and males with patellofemoral pain during functional tasks. The diagram summarizes the number of records identified, screened, assessed for eligibility and included in the qualitative and quantitative synthesis, as well as reasons for exclusion at each stage. PRISMA, Preferred Reporting Items for Systematic Reviews and Meta‐Analyses.

The initial database search yielded 1923 records, of which 172 underwent full‐text screening. Five studies met all inclusion criteria and were included in the final analysis (Figure 1) [3, 21, 27, 28, 44]. Figure 1 presents the PRISMA flow diagram outlining the study selection process.

### Study characteristics

The included studies comprised 170 individuals with PFP (84 females and 86 males), with mean participant ages ranging from 20.9 to 24.7 years. All studies used consistent diagnostic criteria based on insidious onset of PFP unrelated to trauma, pain during functional tasks and symptom duration >3 months.

Tasks assessed included single‐leg squats (*n* = 2) [27, 44], step‐downs (*n* = 1) [28], bilateral mini‐squats (*n* = 1) [21], single‐leg vertical jump (*n* = 1) [31] and single‐leg horizontal jump (*n* = 1) [31]. Kinematic variables included hip adduction, internal rotation, pelvic drop, knee abduction, femoral and tibial adduction, trunk lean and sagittal excursion. Kinetic outcomes were reported in two studies [21, 44]. Motion capture methodologies varied: three‐dimensional (3D) optical (*n* = 2) [3, 31], two‐dimensional photogrammetry (*n* = 1) [21] and 3D electromagnetic systems (*n* = 2) [27, 28], contributing to methodological heterogeneity across studies (Table 2).

**Table 2:** Characteristics of the included studies: Study design, population characteristics, task description and motion capture methods.

Authors (year)	Study design	Participants (PFP)						Task	Task description	Motion capture and data analysis
		**Age (years) (mean ± SD)**	**Sex (*n*) (mean ± SD)**		**Mass (kg) (mean ± SD)**	**Height (m) (mean ± SD)**	**BMI (kg/m^2^) (mean ± SD)**			
Willy et al. 2012 [44]	Cross‐ sectional	M: 24.7 ± 4.9 F: 22.2 ± 3.8	18 F	18 M	NR	NR	M: 25.7 ± 2.0 F: 21.8 ± 2.8	Single‐leg squat	Squat to approximately 60° of knee flexion while standing on the centre of the force platform. Arm position of approximately 90° of shoulder abduction. 1‐Hz count to standardize the speed of the squatting manoeuvre.	8‐camera Vicon Mx system (VICON), 200 Hz. Force platform (Bertec), 1000 Hz.
Nakagawa et al. 2012 [27]	Cross‐ sectional	M: 24.2 ± 4.4 F: 22.3 ± 3.1	20 F	20 M	M: 77.0 ± 9.6 F: 61.1 ± 7.5	M: 1.80 ± 0.05 F: 1.66 ± 0.06	NR	Single‐leg squat	Squat to an angle greater than 60° of knee flexion during a 2‐s period. Return to the initial single leg‐stance position over another 2‐s period. Monitored by a digital metronome, to perform a single‐leg squat.	Flock of Birds tracking device (miniBIRD; Ascension Technology Corporation) in conjunction with MotionMonitor software (Innovative Sports Training, Inc), 90 Hz.
Nakagawa et al. 2013 [28]	Cross‐ sectional	M: 23.6 ± 3.6 F: 22.4 ± 3.2	20 F	20 M	M: 76.0 ± 9.2 F: 61.6 ± 7.8	M: 1.81 ± 0.05 F: 1.66 ± 0.06	NR	Step down	Subjects were instructed to lower themselves from an adjustable step (10% of total body height) over a 2 s period, touch their heel on the floor and return to the starting position over a 2 s period. A metronome was used to control the step rate (15 steps/min).	Flock of Birds® (miniBird®) hardware (Ascension Technology Corporation) loaded with MotionMonitorTM software (Innovative Sports Training, Inc.), 90 Hz.
Hassan et al. 2023 [21]	Cross‐ sectional	20.9 ± 1.4	17 F	13 M	69.1 ± 11.2	1.69 ± 0.08	24.2 ± 3.6	Bilateral mini‐squat	Bilateral squat to 60° of knee flexion. Metronome at 60 bpm. Subjects were instructed to move down on a beat and up on a beat.	Two‐dimensional (2D) photogrammetric analysis using a digital camera (iPhone 6 camera, 8‐megapixel sensor, f/2.2 aperture, 29 mm, 1/3″, 1.5 µm).
Pan et al. 2025 [31]	Cross‐ sectional	M: 22.7 ± 2.3 F: 22.9 ± 4.7	9 F	15 M	M: 67.3 ± 6.2 F: 58.0 ± 7.6	M: 1.76 ± 0.06 F: 1.64 ± 0.04	M: 21.7 ± 1.6 F: 21.5 ± 2.8	SLHJ, SLVJ	With both hands on hips on the force plate, participants were asked to perform amax‐effortt vertical or horizontal jump from a single‐legged stance and land on the same leg (SLHJ landed on separate force plate).	8‐camera motion capture system (Qualisys Arqus12, 200 Hz). Force plates (Kistler, 1000 Hz)

Abbreviations: BMI, body mass index; F, female; M, male; NR, not reported; PFP, patellofemoral pain; SD, standard deviation; SLHJ, single‐leg horizontal jump; SLVJ, single‐leg vertical jump.

### Methodological quality and risk of bias in individual studies

The overall quality rating for each study ranged from moderate to high quality (HQ; Supporting Information S1: Material 3). All studies mitigated the risk of bias by having a well‐defined research question, pre‐specified eligibility criteria, a clear description of outcome measurements and results and consideration for key confounding variables, among others. Willy et al. [44], Nakagawa et al. [27] and Nakagawa et al. [28] were rated as HQ, while Hassan et al. [21] and Pan et al. [31] were rated as moderate quality (MQ).

### Risk of bias of the systematic review

Risk of bias was assessed using the ROBIS tool [42], which evaluates four domains: Domain 1 (study eligibility criteria), Domain 2 (identification and selection of studies), Domain 3 (data collection and study appraisal) and Domain 4 (synthesis and findings). Low concern regarding bias was identified in Domains 1–3, whereas Domain 4 demonstrated unclear concern regarding bias due to the limited number of included studies and the restricted assessment of publication bias. Overall, the systematic review was judged to have a low risk of bias. The complete ROBIS assessment is available in Supporting Information S1: Material 4.

## MAIN RESULTS

The kinetic and kinematic results of all evaluated studies are detailed in Table 3.

**Table 3:** Kinematic and kinetic outcomes comparing females and males with patellofemoral pain during functional tasks.

Kinematic outcomes, Authors (year)	Task	Females (mean ± SD) (°)	Males (mean ± SD) (°)	*p*
Mean sagittal trunk excursion				
Hassan et al. 2023 [21]	Mini‐squat	8.5 ± 3.1	15.3 ± 5.6	0.01*
Ipsilateral trunk lean				
Nakagawa et al. 2012 [27]	Single‐leg squat	11.1 ± 4.6	7.5 ± 3.9	>0.05
Peak contralateral pelvic drop				
Willy et al. 2012 [44]	Single‐leg squat	1.7 ± 3.3	1.3 ± 3.8	0.733
Nakagawa et al. 2012 [27]	Single‐leg squat	11.3 ± 4.3	9.2 ± 4.6	>0.05
Peak hip adduction				
Willy et al. 2012 [44]	Single‐leg squat	10.4 ± 4.2	6.2 ± 4.4	0.007*
Nakagawa et al. 2012 [27]	Single‐leg squat	20.4 ± 6.0	13.9 ± 7.3	>0.05
Nakagawa et al. 2013 [28]	Step down	20.8 ± 4.1	15.4 ± 4.1	0.008*
Peak hip internal rotation				
Willy et al. 2012 [44]	Single‐leg squat	5.2 ± 5.5	5.8 ± 5.4	0.745
Nakagawa et al. 2012 [27]	Single‐leg squat	15.6 ± 5.8	9.8 ± 4.8	0.02*
Nakagawa et al. 2013 [28]	Step down	11.5 ± 4.3	7.5 ± 3.9	0.03*
Peak hip flexion				
Pan et al. 2025 [31]	SLHJ—Propulsion	53.1 ± 7.9	69.5 ± 12.4	0.0001
	SLHJ—Landing	61.5 ± 10.3	58.3 ± 9.2	0.615
	SLVJ—Propulsion	50.0 ± 8.1	60.7 ± 15.0	0.022
	SLVJ—Landing	35.5 ± 13.9	33.6 ± 13.7	0.500
Peak femoral adduction				
Willy et al. 2012 [44]	Single‐leg squat	11.4 ± 2.1	8.4 ± 2.8	0.001*
Peak knee flexion				
Pan et al. 2025 [31]	SLHJ—Propulsion	56.4 ± 4.8	65.3 ± 8.2	0.0001
	SLHJ—Landing	60.9 ± 12.6	57.5 ± 11.9	0.748
	SLVJ—Propulsion	62.7 ± 5.5	69.6 ± 11.7	0.025
	SLVJ—Landing	52.5 ± 8.4	48.8 ± 12.1	0.771
Peak knee abduction				
Willy et al. 2012 [44]	Single‐leg squat	1.6 ± 4.8	−6.0 ± 4.6	<0.001*
Nakagawa et al. 2012 [27]	Single‐leg squat	11.2 ± 4.6	7.1 ± 3.5	>0.05
Nakagawa et al. 2013 [28]	Step down	10.9 ± 4.1	6.8 ± 3.2	0.007*
Peak tibial adduction				
Willy et al. 2012 [44]	Single‐leg squat	3.8 ± 2.7	6.3 ± 2.0	0.004*
Peak ankle flexion				
Pan et al. 2025 [31]	SLHJ—Propulsion	31.8 ± 7.0	33.6 ± 4.1	0.715
	SLHJ—Landing	10.0 ± 8.1	11.9 ± 6.2	0.512
	SLVJ—Propulsion	26.6 ± 6.6	25.1 ± 4.0	0.750
	SLVJ—Landing	23.8 ± 7.7	19.7 ± 3.7	0.534

Kinetic outcomes, Authors (year)		Females (mean ± SD) (N·m·kg^−1^·m^−1^)	Males (mean ± SD) (N·m·kg^−1^·m^−1^)	*p*
Peak hip extension moment				
Pan et al. 2025 [31]	SLHJ—Propulsion	−0.156 ± 0.349	−0.214 ± 0.045	0.001*
	SLHJ—Landing	−0.342 ± 0.073	−0.369 ± 0.185	0.203
	SLVJ—Propulsion	−0.104 ± 0.29	−0.161 ± 0.041	0.0001*
	SLVJ—Landing	−0.094 ± 0.037	−0.168 ± 0.072	0.002*
Peak external knee adduction moment				
Willy et al. 2012 [44]	Single‐leg squat	0.184 ± 0.079	0.220 ± 0.073	0.165
Peak knee extension moment				
Pan et al. 2025 [31]	SLHJ—Propulsion	−0.102 ± 0.034	−0.113 ± 0.03	0.015*
	SLHJ—Landing	−0.175 ± 0.024	−0.212 ± 0.028	0.021*
	SLVJ—Propulsion	−0.139 ± 0.03	−0.155 ± 0.043	0.056
	SLVJ—Landing	−0.151 ± 0.03	−0.179 ± 0.039	0.027*
Peak ankle extension moment				
Pan et al. 2025 [31]	SLHJ—Propulsion	−0.150 ± 0.033	−0.155 ± 0.025	0.105
	SLHJ—Landing	−0.067 ± 0.034	−0.097 ± 0.038	0.019*
	SLVJ—Propulsion	−0.136 ± 0.028	−0.162 ± 0.036	0.01*
	SLVJ—Landing	−0.137 ± 0.033	−0.156 ± 0.04	0.025*

*Note*: Data are presented as mean ± SD for each biomechanical variable. Reported outcomes include trunk, pelvic, hip, knee and ankle kinematics and kinetics assessed during squatting and jumping tasks. Significant between‐sex differences are indicated by *p* values < 0.05. Negative values reflect the coordinate‐system and sign conventions adopted in the original studies.

Abbreviations: SD, standard deviation; SLHJ, single‐leg horizontal jump; SLVJ, single‐leg vertical jump.

* *p *< 0.05

### KINEMATICS

#### Trunk

Hassan et al. [21] reported increased mean sagittal trunk excursion in males compared to females during a mini‐squat, with a mean difference of 6.8° (*p* = 0.01). Nakagawa et al. [27] reported a greater peak ipsilateral trunk lean in females during a single‐leg squat; however, without reaching statistical significance (*p* > 0.05) (Table 3).

#### Pelvic

Peak contralateral pelvic drop was reported in two studies [27, 44]. Willy et al. [44] and Nakagawa et al. [27] reported greater peak contralateral pelvic drop in females with PFP compared to males with PFP during a single‐leg squat, but neither study found statistically significant differences between the two cohorts (Table 3).

**Figure 2:**
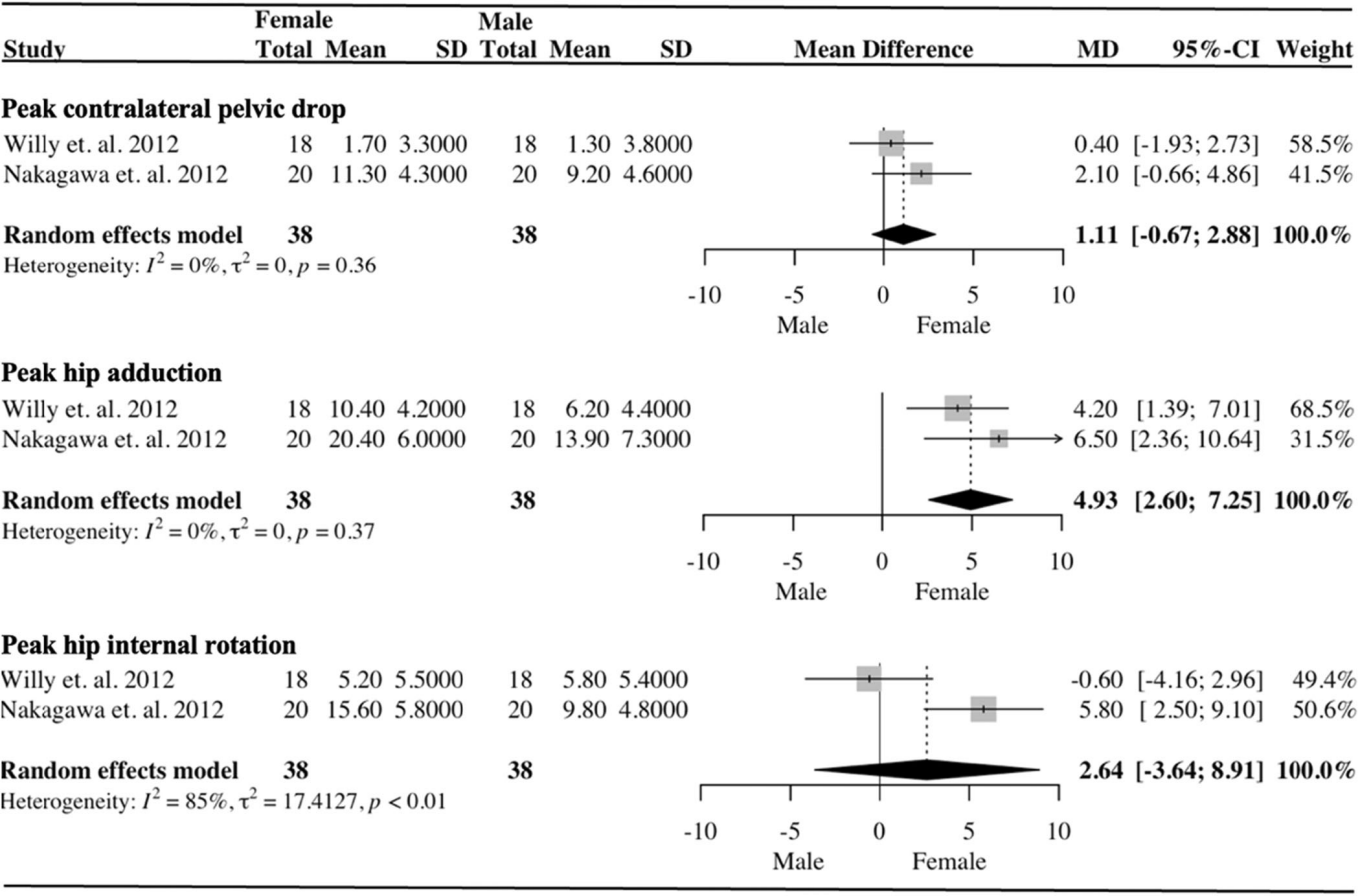
Forest plots of meta‐analyses comparing peak hip adduction and peak hip internal rotation between females and males with patellofemoral pain during the single‐leg squat task. Positive mean differences indicate greater values in females compared with males. Squares represent individual study effect sizes weighted by sample size, and diamonds represent pooled estimates with 95% CIs. CI, confidence interval; MD, mean difference; SD, standard deviation.

A meta‐analysis was performed by pooling the data from two studies that examined identical kinematic variables of the peak contralateral pelvic drop during the single‐leg squat [27, 44] (Figure 2). The mean difference was 1.11 with a 95% CI of [−0.67, 2.88], indicating no statistically significant difference between sexes (*p* > 0.05). The heterogeneity was low (*I* ^2 ^= 0%, *τ* ^2 ^= 0, *p* = 0.36).

#### Hip

Three studies reported higher peak hip adduction [27, 28, 44] in females compared to males; however, only in two of them [28, 44] the differences were statistically significant (Table 3). Nakagawa et al. [27] and Nakagawa et al. [28] reported significantly greater peak hip internal rotation in females, with a mean difference of 5.8° and 4° during a single‐leg squat and step‐down task, respectively. In a single‐leg squat task, Willy et al. [44] reported no differences between females and males.

Regarding hip flexion outcomes, task‐dependent sex differences were observed. During the propulsion phase of the single‐leg horizontal jump, females demonstrated significantly lower hip flexion compared with males (53.1 ± 7.9° vs. 69.5 ± 12.4°, *p* = 0.0001). A similar pattern was identified during the propulsion phase of the single‐leg vertical jump, with lower hip flexion values in females (50.0 ± 8.1°, *p* = 0.022) than in males (60.7 ± 15.0°). In contrast, no statistically significant sex differences were observed during the landing phase, either for the single‐leg horizontal jump (females: 61.5 ± 10.3°; males: 58.3 ± 9.2°, *p* = 0.615) or the single‐leg vertical jump (35.5 ± 13.9° vs. 33.6 ± 13.7°, *p* = 0.500).

A meta‐analysis was performed by pooling data from two studies that examined identical kinematic variables of the hip adduction and hip internal rotation during the single‐leg squat [27, 44] (Figure 2). For peak hip adduction, the meta‐analysis showed a pooled mean difference of 4.93 with a 95% CI of [2.60, 7.25], reflecting significantly greater peak hip adduction in females compared to males. Heterogeneity was low (*I* ^2 ^= 0%, *τ* ^2 ^= 0, *p* = 0.37). For peak hip internal rotation, the pooled mean difference was 2.64 with a 95% CI of [−3.64, 8.91], which did not represent a statistically significant difference between sexes. However, substantial heterogeneity was observed (*I* ^2 ^= 85%, *τ* ^2 ^= 17.41, *p* < 0.01).

#### Knee

When examining knee flexion outcomes, sex‐specific differences were also task‐ and phase‐dependent. During the propulsion phase of the single‐leg horizontal jump, females exhibited significantly lower knee flexion compared with males (56.4 ± 4.8° vs. 65.3 ± 8.2°, *p* = 0.0001). Similarly, during the propulsion phase of the single‐leg vertical jump, females exhibited lower knee flexion than males (62.7 ± 5.5° vs. 69.6 ± 11.7°, *p* = 0.025). In contrast, no statistically significant sex differences were observed during the landing phase of either task, including the single‐leg horizontal jump (females: 60.9 ± 12.6°; males: 57.5 ± 11.9°, *p* = 0.748) or the single‐leg vertical jump (52.5 ± 8.4° vs. 48.8 ± 12.1°, *p *= 0.771).

Peak knee abduction was reported in three studies [27, 28, 44]. Willy et al. [44] and Nakagawa et al. [27] found significantly greater knee abduction in females and a mean difference of 7.6° and 4.1°, in a single‐leg squat and a step‐down task, respectively. Nakagawa et al. [28], in a single‐leg squat task, did not find significant differences between females and males. Data pooling was performed with two studies that examined peak knee abduction during the single‐leg squat. Only Willy et al. [44] reported both femoral and tibial adduction in a single‐leg squat. Peak femoral adduction was greater in females, while peak tibial adduction was higher in males, both statistically significant.

**Figure 3:**
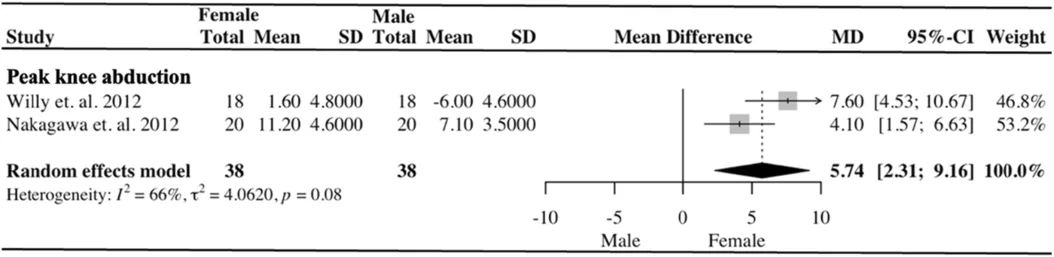
Forest plot of the meta‐analysis comparing peak knee abduction between females and males with patellofemoral pain during the single‐leg squat task. Positive mean differences indicate greater knee abduction values in females compared with males. Squares represent individual study effect sizes weighted by sample size, and diamonds represent pooled estimates with 95% CIs. CI, confidence interval; MD, mean difference; SD, standard deviation.

Data pooling was performed with two studies that examined peak knee abduction during the single‐leg squat [27, 44] (Figure 3). The pooled mean difference in peak knee abduction between sexes was 5.74, with a 95% CI of [2.31, 9.16]. This indicates that females had a statistically significantly higher peak knee abduction than males across the studies included. The heterogeneity of the effect sizes was moderate to substantial (*I* ^2 ^= 66%, *τ* ^2 ^= 4.0620, *p* = 0.08).

#### Ankle

Regarding ankle flexion outcomes, no statistically significant sex differences were observed across tasks or phases; however, this variable was evaluated in only one study [31], which limits the strength of this finding. During the propulsion phase of the single‐leg horizontal jump, ankle flexion values were comparable between females (31.8 ± 7.0°) and males (33.6 ± 4.1°, *p* = 0.715). Similarly, during landing, no significant difference was identified between females (10.0 ± 8.1°) and males (11.9 ± 6.2°, *p* = 0.512). Consistent findings were observed during the single‐leg vertical jump, with no sex‐related differences during propulsion (females: 26.6 ± 6.6°; males: 25.1 ± 4.0°, *p* = 0.750) or landing (23.8 ± 7.7° vs. 19.7 ± 3.7°, *p* = 0.534).

### KINETICS

#### Hip

Hip kinetic outcomes were reported exclusively by Pan et al. [31]. Significant sex differences were observed for the peak hip extension moment during several jumping phases; nevertheless, this result is based on a single study. During the propulsion phase of the single‐leg horizontal jump, females demonstrated significantly lower hip extension moments compared with males (−0.156 ± 0.349 vs. −0.214 ± 0.045 N·m·kg^−1^·m^−1^; *p* = 0.001). A similar pattern was observed during the propulsion phase of the single‐leg vertical jump, with lower values in females (−0.104 ± 0.29) compared with males (−0.161 ± 0.041; *p* = 0.0001). During landing, no significant sex difference was identified for the single‐leg horizontal jump (*p* = 0.203); however, females exhibited significantly lower hip extension moments during single‐leg vertical jump landing (−0.094 ± 0.037 vs. −0.168 ± 0.072; *p* = 0.002) (Table 3).

#### Knee

Knee kinetic outcomes were reported by Willy et al. [44] and Pan et al. [31]. For the peak external knee adduction moment assessed during a single‐leg squat, Willy et al. [44] found no statistically significant difference between females (0.184 ± 0.079 N·m·kg^−1^·m^−1^) and males (0.220 ± 0.073 N·m·kg^−1^·m^−1^; *p* = 0.165).

Regarding sagittal‐plane kinetics, Pan et al. [31] reported sex differences in the peak knee extension moment across several jumping phases. Females demonstrated significantly lower knee extension moments during single‐leg horizontal jump propulsion (−0.102 ± 0.034 vs. −0.113 ± 0.03; *p* = 0.015), single‐leg horizontal jump landing (−0.175 ± 0.024 vs. −0.212 ± 0.028; *p* = 0.021) and single‐leg vertical jump landing (−0.151 ± 0.03 vs. −0.179 ± 0.039; *p* = 0.027). No statistically significant difference was observed during single‐leg vertical jump propulsion (*p* = 0.056).

#### Ankle

Ankle kinetic outcomes were reported exclusively by Pan et al. [31]. For the peak ankle extension moment, no significant sex difference was observed during single‐leg horizontal jump propulsion (females: 0.150 ± 0.033; males: 0.155 ± 0.025 N·m·kg^−1^·m^−1^; *p* = 0.105). In contrast, females exhibited significantly lower ankle extension moments during single‐leg horizontal jump landing (−0.067 ± 0.034 vs. −0.097 ± 0.038; *p* = 0.019), single‐leg vertical jump propulsion (−0.136 ± 0.028 vs. −0.162 ± 0.036; *p* = 0.01) and landing (−0.137 ± 0.033 vs. −0.156 ± 0.04; *p* = 0.025).

## DISCUSSION

This systematic review synthesized kinematic and kinetic evidence comparing females and males with PFP across a range of functional tasks, including squatting, step‐downs and single‐leg jumping activities. Two main findings emerged from this review. First, only five studies met the inclusion criteria, highlighting the limited body of evidence available on sex‐specific biomechanical differences in individuals with PFP. This small number of investigations restricts the strength of the conclusions and limits the ability to perform robust comparisons across tasks and outcomes. Second, despite the limited evidence, consistent biomechanical differences between females and males with PFP were observed across functional activities, suggesting that sex may influence movement strategies in this population and should be considered when evaluating patients with PFP.

Abnormal biomechanics have been established as a typical kinematic pattern observed in individuals with PFP [34]; however, the present study demonstrated that movement behaviour differs between females and males across a range of functional tasks. The present study found that females exhibited greater peak hip adduction and knee abduction compared with males. Additionally, the qualitative analysis showed that these differences may extend to hip internal rotation, femoral adduction and tibial abduction. Although the overall number of studies evaluating sexes separately remains limited, the studies included in this review provide valuable insights into potential sex‐specific patterns in lower limb kinematics.

Kinetic findings indicated that males demonstrated greater extension moments at the hip, knee and ankle during jumping tasks, particularly during propulsion, whereas females may adopt strategies characterized by reduced moments. Nonetheless, these kinetic outcomes were also reported in a limited number of studies, often based on isolated evidence, which weakens the strength of these observations and limits the ability to draw definitive conclusions. A recent systematic review by Hoglund et al. [24] found that muscle weakness patterns in males with PFP differed from those in females. Specifically, males with PFP showed weakness in the hip extensors, but unlike females with PFP, the majority of them did not demonstrate weakness in the hip abductors or external rotators. These findings support the presence of sex‐specific biomechanical characteristics and suggest that rehabilitation strategies may need to be individualized according to sex.

Regarding the functional tasks evaluated, two studies utilized the single‐leg squat, one study evaluated the step‐down, and another study evaluated the bilateral mini‐squat. Notably, there was significant heterogeneity in task selection and kinematic assessment methods across studies. Even within the single‐leg squat group, studies measured kinematic parameters at different movement phases, hindering direct comparisons. This lack of standardization identified in our study aligns with prior reports in the literature [41]. This inconsistency represents a hindrance to generating robust evidence in biomechanics and emphasizes the importance of standardization as a major concern for future studies. However, despite methodological differences, similar directional findings were observed, which strengthens the clinical relevance of sex‐specific biomechanical patterns.

Due to the cross‐sectional design of the studies included in this systematic review, we cannot make inferences about the causal relationship between the biomechanical changes observed and the presence of PFP. However, prospective evidence supports the presence of sex‐specific risk factors. Boling et al. [3] evaluated a large cohort of 3893 healthy cadets and identified distinct predictors for the development of PFP in females and males during a jump‐landing task. Females who later developed PFP demonstrated less hip abduction at initial contact and greater knee internal rotation during stance, whereas males exhibited increased risk when landing with decreased knee flexion and increased hip external rotation. These prospective findings align with the sex‐specific biomechanical patterns identified in the present review and further support the importance of considering sex differences when evaluating movement mechanics and designing prevention strategies.

In light of these considerations, this study emphasizes the significance of accounting for sex‐based biomechanical differences in the clinical management of PFP. The observed distinct kinematic patterns between females and males with this condition suggest that a ‘one‐size‐fits‐all’ approach may be inadequate. However, the cross‐sectional design of the studies does not allow inferences about causal relationships between the observed biomechanical changes and the presence of PFP.

This review has limitations that should be acknowledged. First, the small number of studies available for quantitative synthesis limited the robustness of pooled estimates. Most meta‐analyses included only two studies, which limits the robustness of pooled estimates, reduces the reliability of heterogeneity assessment and prevents meaningful evaluation of publication bias. Consequently, the quantitative findings should be interpreted as exploratory. Additionally, the limited number of studies and relatively small sample sizes restrict the external validity and generalizability of the results. Second, most of the included studies employed cross‐sectional designs, which preclude causal inferences regarding the relationship between biomechanical alterations and the presence of PFP. Additionally, two included studies were conducted at the same research centre, raising the possibility of overlapping participants. However, the studies evaluated different functional tasks, which reduces the likelihood of duplicated biomechanical outcomes. Nevertheless, potential sample overlap cannot be excluded and should be considered when interpreting the results. Finally, substantial methodological heterogeneity was observed across studies, including differences in task selection and kinematic data acquisition and processing, which may have influenced the pooled results and limited the generalizability of the findings.

Future studies should prioritize standardized task selection, consistent reporting of kinematic and kinetic variables and inclusion of sex‐stratified analyses. In addition, prospective and longitudinal investigations are needed to clarify causal mechanisms and determine whether sex‐specific biomechanical patterns contribute to the development and persistence of PFP. Expanding research in male populations is particularly important, as current evidence remains limited.

## CONCLUSION

In conclusion, this systematic review identified a limited number of studies investigating sex‐specific biomechanics in individuals with PFP. Despite this restricted evidence base, consistent differences between females and males were observed across functional tasks. These findings suggest that sex influences movement mechanics in PFP and should be considered in both biomechanical assessment and clinical management.

## AUTHOR CONTRIBUTIONS

*Conceptualization*: Lucas Pallone, Felipe F. Gonzalez, Leonardo Metsavaht and Gustavo Leporace. *Data curation*: Talissa O. Generoso, Rodrigo Saad Berreta, Kaitlyn M. Joyce, Carolline P. Nunes, Eliane C. Guadagnin, Jorge Chahla and Jonathan A. Gustafson. *Formal analysis*: Lucas Pallone, Carolline P. Nunes, Felipe F. Gonzalez, Leonardo Metsavaht and Gustavo Leporace. *Investigation*: Lucas Pallone, Carolline P. Nunes, Felipe F. Gonzalez, Leonardo Metsavaht and Gustavo Leporace. *Methodology*: Lucas Pallone, Carolline P. Nunes, Felipe F. Gonzalez, Leonardo Metsavaht and Gustavo Leporace. *Project administration*: Lucas Pallone, Carolline P. Nunes and Felipe F. Gonzalez. *Supervision*: Lucas Pallone, Carolline P. Nunes, Felipe F. Gonzalez, Leonardo Metsavaht and Gustavo Leporace. *Validation, visualization and writing—original draft*: Lucas Pallone, Carolline P. Nunes, Felipe F. Gonzalez, Talissa O. Generoso, Rodrigo Saad Berreta, Kaitlyn M. Joyce, Carolline P. Nunes, Eliane C. Guadagnin, Jorge Chahla, Jonathan A. Gustafson, Leonardo Metsavaht and Gustavo Leporace. *Writing—review and editing*: Lucas Pallone, Carolline P. Nunes, Felipe F. Gonzalez, Talissa O. Generoso, Rodrigo Saad Berreta, Kaitlyn M. Joyce, Eliane C. Guadagnin, Jorge Chahla, Jonathan A. Gustafson, Leonardo Metsavaht and Gustavo Leporace.

## FUNDING INFORMATION

This study was supported by Instituto Brasil de Tecnologias da Saúde (IBTS) and financed in part by the Coordenação de Aperfeiçoamento de Pessoal de Nível Superior—Brasil (CAPES)—Finance Code 001.

## CONFLICT OF INTEREST STATEMENT

Jorge Chahla is a board member of the American Orthopaedic Society for Sports Medicine, the Arthroscopy Association of North America and the International Society of Arthroscopy, Knee Surgery and Orthopaedic Sports Medicine; consultant/advisory role for Arthrex Inc., CONMED Corp., Ossur Americas and Smith & Nephew Inc.; received speaking and lecture fees from Smith & Nephew Inc. The remaining authors declare no conflicts of interest.

## ETHICS STATEMENT

The authors have nothing to report.

## Supporting information

Supplementary Information

## Data Availability

The data that support the findings of this study are available from the corresponding author upon reasonable request.
