# Supplementary material for: Biomechanical differences between females and males with patellofemoral pain: A systematic review and meta‐analysis
Source: J Exp Orthop. 2026 Aug 21;13(3):e70880. doi: 10.1002/jeo2.70880 (PMC13495770; doi:10.1002/jeo2.70880)
Supplement: Supplementary file 1 [file JEO2-13-e70880-s001.docx]

**Supplementary Material 1**

PRISMA 2020 checklist

| **Section and Topic** | **Item #** | **Checklist item** | **Location where item is reported** |
| --- | --- | --- | --- |
| **TITLE** | | |  |
| Title | 1 | Identify the report as a systematic review. | Title page |
| **ABSTRACT** | | |  |
| Abstract | 2 | See the PRISMA 2020 for Abstracts checklist. | Abstract |
| **INTRODUCTION** | | |  |
| Rationale | 3 | Describe the rationale for the review in the context of existing knowledge. | Introduction, paragraphs 1–3 |
| Objectives | 4 | Provide an explicit statement of the objective(s) or question(s) the review addresses. | End of Introduction |
| **METHODS** | | |  |
| Eligibility criteria | 5 | Specify the inclusion and exclusion criteria for the review and how studies were grouped for the syntheses. | Methods — Eligibility Criteria section |
| Information sources | 6 | Specify all databases, registers, websites, organisations, reference lists and other sources searched or consulted to identify studies. Specify the date when each source was last searched or consulted. | Methods — Search methods and study selection |
| Search strategy | 7 | Present the full search strategies for all databases, registers and websites, including any filters and limits used. | Table 1 + Supplementary material |
| Selection process | 8 | Specify the methods used to decide whether a study met the inclusion criteria of the review, including how many reviewers screened each record and each report retrieved, whether they worked independently, and if applicable, details of automation tools used in the process. | Methods — Search methods and study selection |
| Data collection process | 9 | Specify the methods used to collect data from reports, including how many reviewers collected data from each report, whether they worked independently, any processes for obtaining or confirming data from study investigators, and if applicable, details of automation tools used in the process. | Methods — Data Extraction |
| Data items | 10a | List and define all outcomes for which data were sought. Specify whether all results that were compatible with each outcome domain in each study were sought (e.g. for all measures, time points, analyses), and if not, the methods used to decide which results to collect. | Methods — Data Extraction |
|  | 10b | List and define all other variables for which data were sought (e.g. participant and intervention characteristics, funding sources). Describe any assumptions made about any missing or unclear information. | Methods — Data Extraction |
| Study risk of bias assessment | 11 | Specify the methods used to assess risk of bias in the included studies, including details of the tool(s) used, how many reviewers assessed each study and whether they worked independently, and if applicable, details of automation tools used in the process. | Methods — Risk of bias in individual studies |
| Effect measures | 12 | Specify for each outcome the effect measure(s) (e.g. risk ratio, mean difference) used in the synthesis or presentation of results. | Methods — Data Analysis |
| Synthesis methods | 13a | Describe the processes used to decide which studies were eligible for each synthesis (e.g. tabulating the study intervention characteristics and comparing against the planned groups for each synthesis (item #5)). | Methods — Data Analysis |
|  | 13b | Describe any methods required to prepare the data for presentation or synthesis, such as handling of missing summary statistics, or data conversions. | Methods — Data Analysis |
|  | 13c | Describe any methods used to tabulate or visually display results of individual studies and syntheses. | Tables 2–3 and Figures 2–3 |
|  | 13d | Describe any methods used to synthesize results and provide a rationale for the choice(s). If meta-analysis was performed, describe the model(s), method(s) to identify the presence and extent of statistical heterogeneity, and software package(s) used. | Methods — Data Analysis |
|  | 13e | Describe any methods used to explore possible causes of heterogeneity among study results (e.g. subgroup analysis, meta-regression). | Methods — Data Analysis |
|  | 13f | Describe any sensitivity analyses conducted to assess robustness of the synthesized results. | Sensitivity analyses were not performed due to the small number of included studies. |
| Reporting bias assessment | 14 | Describe any methods used to assess risk of bias due to missing results in a synthesis (arising from reporting biases). | Methods — Risk of Bias Across Studies |
| Certainty assessment | 15 | Describe any methods used to assess certainty (or confidence) in the body of evidence for an outcome. | Certainty of evidence was not assessed due to the small number and heterogeneity of studies. |
| **RESULTS** | | |  |
| Study selection | 16a | Describe the results of the search and selection process, from the number of records identified in the search to the number of studies included in the review, ideally using a flow diagram. | Results — Study selection + Figure 1 |
|  | 16b | Cite studies that might appear to meet the inclusion criteria, but which were excluded, and explain why they were excluded. | Results — Study selection + Figure 1 |
| Study characteristics | 17 | Cite each included study and present its characteristics. | Results — Study Characteristics + Table 2 |
| Risk of bias in studies | 18 | Present assessments of risk of bias for each included study. | Results — Risk of bias in individual studies + Table 4 |
| Results of individual studies | 19 | For all outcomes, present, for each study: (a) summary statistics for each group (where appropriate) and (b) an effect estimate and its precision (e.g. confidence/credible interval), ideally using structured tables or plots. | Results — Kinematics and Kinetics + Table 3 |
| Results of syntheses | 20a | For each synthesis, briefly summarise the characteristics and risk of bias among contributing studies. | Results — meta-analysis description |
|  | 20b | Present results of all statistical syntheses conducted. If meta-analysis was done, present for each the summary estimate and its precision (e.g. confidence/credible interval) and measures of statistical heterogeneity. If comparing groups, describe the direction of the effect. | Results + Figures 2–3 |
|  | 20c | Present results of all investigations of possible causes of heterogeneity among study results. | Results — meta-analysis sections |
|  | 20d | Present results of all sensitivity analyses conducted to assess the robustness of the synthesized results. | Sensitivity analyses were not performed due to the small number of included studies. |
| Reporting biases | 21 | Present assessments of risk of bias due to missing results (arising from reporting biases) for each synthesis assessed. | Results — Risk of bias across studies |
| Certainty of evidence | 22 | Present assessments of certainty (or confidence) in the body of evidence for each outcome assessed. | Certainty of evidence was not assessed due to the small number and heterogeneity of studies. |
| **DISCUSSION** | | |  |
| Discussion | 23a | Provide a general interpretation of the results in the context of other evidence. | Discussion |
|  | 23b | Discuss any limitations of the evidence included in the review. | Discussion — limitations paragraph |
|  | 23c | Discuss any limitations of the review processes used. | Discussion — heterogeneity + small number of studies |
|  | 23d | Discuss implications of the results for practice, policy, and future research. | Discussion — final paragraphs |
| **OTHER INFORMATION** | | |  |
| Registration and protocol | 24a | Provide registration information for the review, including register name and registration number, or state that the review was not registered. | Methods — Manuscript Registration (PROSPERO) |
|  | 24b | Indicate where the review protocol can be accessed, or state that a protocol was not prepared. | Methods — Manuscript Registration |
|  | 24c | Describe and explain any amendments to information provided at registration or in the protocol. | Not applicable |
| Support | 25 | Describe sources of financial or non-financial support for the review, and the role of the funders or sponsors in the review. | Acknowledgments (blinded) |
| Competing interests | 26 | Declare any competing interests of review authors. | To be declared in journal submission system |
| Availability of data, code and other materials | 27 | Report which of the following are publicly available and where they can be found: template data collection forms; data extracted from included studies; data used for all analyses; analytic code; any other materials used in the review. | All data extracted from included studies are available within the manuscript and supplementary material. |

**PRISMA 2020 for Abstracts Checklist**

| **Section and Topic** | **Item #** | **Checklist item** | **Reported (Yes/No)** |
| --- | --- | --- | --- |
| **TITLE** | | |  |
| Title | 1 | Identify the report as a systematic review. | Yes |
| **BACKGROUND** | | |  |
| Objectives | 2 | Provide an explicit statement of the main objective(s) or question(s) the review addresses. | Yes |
| **METHODS** | | |  |
| Eligibility criteria | 3 | Specify the inclusion and exclusion criteria for the review. | Yes |
| Information sources | 4 | Specify the information sources (e.g. databases, registers) used to identify studies and the date when each was last searched. | Yes |
| Risk of bias | 5 | Specify the methods used to assess risk of bias in the included studies. | Yes |
| Synthesis of results | 6 | Specify the methods used to present and synthesise results. | Yes |
| **RESULTS** | | |  |
| Included studies | 7 | Give the total number of included studies and participants and summarise relevant characteristics of studies. | Yes |
| Synthesis of results | 8 | Present results for main outcomes, preferably indicating the number of included studies and participants for each. If meta-analysis was done, report the summary estimate and confidence/credible interval. If comparing groups, indicate the direction of the effect (i.e. which group is favoured). | Yes |
| **DISCUSSION** | | |  |
| Limitations of evidence | 9 | Provide a brief summary of the limitations of the evidence included in the review (e.g. study risk of bias, inconsistency and imprecision). | Not applicable. The journal requires a structured abstract without a Discussion section |
| Interpretation | 10 | Provide a general interpretation of the results and important implications. |  |
| **OTHER** | | |  |
| Funding | 11 | Specify the primary source of funding for the review. | No |
| Registration | 12 | Provide the register name and registration number. | Yes |

*From:* Page MJ, McKenzie JE, Bossuyt PM, Boutron I, Hoffmann TC, Mulrow CD, et al. The PRISMA 2020 statement: an updated guideline for reporting systematic reviews. BMJ 2021;372:n71. doi: 10.1136/bmj.n71. This work is licensed under CC BY 4.0. To view a copy of this license, visit <https://creativecommons.org/licenses/by/4.0/>

**Supplementary Material 2**

Search strategy

N = 1,923

PubMed: 467

Cochrane Database of Systematic Reviews: 11

Cochrane CENTRAL Register of Controlled Trials: 121

Scopus: 460

Embase: 410

CINAHL: 302

Google Scholar: 88

ClinicalTrials.gov: 64

PubMed: 467

(Patellofemoral OR patello-femoral OR femoropatellar OR femoro-patellar OR retropatellar OR "anterior knee" OR "Patellofemoral Joint"[Mesh] OR "Patellofemoral Pain Syndrome"[Mesh])

AND (pain OR syndrome OR dysfunction OR chondromalacia OR "Patellofemoral Pain Syndrome"[Mesh] OR "Musculoskeletal Pain"[Mesh])

AND (Squat OR lunge OR Stair OR Step OR jump OR "Stair Climbing"[Mesh])

AND (biomechanic* OR kinematic* OR kinetic* OR "Biomechanical Phenomena"[Mesh] OR "Mechanical Phenomena"[Mesh] OR "Kinetics"[Mesh])

Cochrane Database of Systematic Reviews: 11

Cochane CENTRAL Register of Controlled Trials: 121

#1 MeSH descriptor: [Patellofemoral Joint] explode all trees 90

#2 MeSH descriptor: [Patellofemoral Pain Syndrome] explode all trees 444

#3 Patellofemoral OR patello-femoral OR femoropatellar OR femoro-patellar OR retropatellar OR "anterior knee" 2192

#4	#1 OR #2 OR #3 2192

#5 MeSH descriptor: [Musculoskeletal Pain] explode all trees 1819

#6 pain OR syndrome OR dysfunction OR chondromalacia 452108

#7	#2 OR #5 OR #6 452555

#8 MeSH descriptor: [Stair Climbing] explode all trees 46

#9 Squat OR lunge OR Stair OR Step OR jump 39363

#10	#8 OR #9 39363

#11 MeSH descriptor: [Biomechanical Phenomena] explode all trees 4498

#12 MeSH descriptor: [Kinetics] explode all trees 4154

#13 MeSH descriptor: [Mechanical Phenomena] explode all trees 19701

#14 biomechanic* OR kinematic* OR kinetic* 26265

#15	#11 OR #12 OR #13 OR #14 37119

#16	#4 AND #7 AND #10 AND #15 148

Scopus: 460

(Patellofemoral OR patello-femoral OR femoropatellar OR femoro-patellar

OR retropatellar OR "anterior knee" OR "patellofemoral joint"

OR "patellofemoral pain syndrome")

AND (pain OR syndrome OR dysfunction OR chondromalacia

OR "patellofemoral pain syndrome" OR "musculoskeletal pain")

AND (squat OR lunge OR stair OR step OR jump OR "stair climbing")

AND (biomechanic* OR kinematic* OR kinetic*

OR "biomechanical phenomena" OR "mechanical phenomena" OR kinetics)

Embase: 410

(patellofemoral:ab,ti OR 'patello femoral':ab,ti OR femoropatellar:ab,ti OR 'femoro patellar':ab,ti OR retropatellar:ab,ti OR 'anterior knee':ab,ti OR 'patellofemoral joint'/exp OR 'patellofemoral pain syndrome'/exp)

AND (pain:ab,ti OR syndrome:ab,ti OR dysfunction:ab,ti OR chondromalacia:ab,ti OR 'musculoskeletal pain'/exp OR 'patellofemoral pain syndrome'/exp)

AND (squat:ab,ti OR lunge:ab,ti OR stair:ab,ti OR step:ab,ti OR jump:ab,ti OR 'stair climbing'/exp)

AND (biomechanic*:ab,ti OR kinematic*:ab,ti OR kinetic*:ab,ti OR 'biomechanics'/exp)

CINAHL: 302

(XB (Patellofemoral OR patello-femoral OR femoropatellar OR femoro-patellar OR retropatellar OR "anterior knee") OR MM "Patellofemoral Pain Syndrome" OR MM "Patellofemoral joint")

AND (XB (pain OR syndrome OR dysfunction OR chondromalacia) OR MM "Patellofemoral Pain Syndrome" OR MM "Pain")

AND (XB (Squat OR lunge OR Stair OR Step OR jump) OR MM "Stair Climbing")

AND (XB (biomechanic* OR kinematic* OR kinetic*) OR MM "Biomechanics" OR MM "Kinematics" OR MM “Kinetics” OR MM “Biomechanical Phenomena”)

Google Scholar: 88

(Patellofemoral OR patello-femoral OR femoropatellar OR femoro-patellar OR retropatellar OR "anterior knee")

AND (pain OR syndrome OR dysfunction OR chondromalacia)

AND (Squat OR lunge OR Stair OR Step OR jump)

AND (biomechanical OR biomechanics OR kinematic OR kinetic)

ClinicalTrials.gov: 64

Search date: April 3, 2026

Search terms:

("patellofemoral pain" OR "anterior knee pain" OR "patellofemoral syndrome")

AND

(biomechanics OR kinematics OR kinetics)

No filters applied.

**Supplementary Material 3**

Modified Downs & Black checklist for methodological quality appraisal.

| **Item** | **Title** | **Description by Downs & Black** | ***Willy et al. 2012***^36^ | ***Nakagawa et al. 2012***^37^ | ***Nakagawa et al. 2013***^41^ | ***Hassan et al. 2023***^42^ | ***Pan et al.***  ***2025***^43^ |
| --- | --- | --- | --- | --- | --- | --- | --- |
| 1 | Aim | Is the hypothesis/aim/objective clearly described? | 1 | 1 | 1 | 1 | 1 |
| 2 | Outcomes | Are the main outcomes to be measured clearly described in the Introduction or Methods section? | 1 | 1 | 1 | 1 | 1 |
| 3 | Participants | Are the characteristics of the patients included in the study clearly described? | 1 | 1 | 1 | 1 | 1 |
| 5 | Confounders | Are distributions of principal confounders in each group of subjects to be compared clearly described? | 2 | 2 | 1 | 0 | 1 |
| 6 | Findings | Are the main findings of the study clearly described? | 1 | 1 | 1 | 1 | 1 |
| 7 | Random variability | Does the study provide estimates of the random variability in the data for the main outcomes | 1 | 1 | 1 | 1 | 1 |
| 10 | Probability | Have actual probability values been reported for the main outcomes except where the probability value in less than 0.001? | 1 | 1 | 1 | 1 | 1 |
| 11 | External validity | Were the subjects asked to participate in the study representative of the entire population from which they were recruited? | 0 | 0 | 0 | 0 | 0 |
| 12 | External validity | Were those subjects who were prepared to participate representative of the entire population from which they were recruited? | 0 | 0 | 0 | 0 | 0 |
| 15 | Blinding | Was an attempt made to blind those measuring the main outcomes of the intervention? | 0 | 0 | 0 | 0 | 0 |
| 18 | Statistical tests | Were the statistical tests used to assess the main outcomes appropriate? | 1 | 1 | 1 | 1 | 1 |
| 20 | Accurate outcomes | Were the main outcome measures used accurate (valid and reliable)? | 1 | 1 | 1 | 0 | 0 |
| 25 | Confounding adjustment | Was there adequate adjustment for confounding in the analyses from which the main findings were drawn? | 1 | 1 | 1 | 0 | 0 |
| 26 | Loss to follow-up | Were losses of patients to follow-up taken into account? | 0 | 0 | 0 | 0 | 0 |
| 27 | Power | Did the study have sufficient power to detect a clinically important effect where probability value for difference being due to chance is < 5% | 1 | 1 | 1 | 1 | 1 |
| **Score** | | | 12 | 12 | 11 | 8 | 9 |
| **Quality** | | | HQ | HQ | HQ | MQ | MQ |

**Supplementary Material 4**

ROBIS assessment

ROBIS (Risk Of Bias In Systematic Reviews) assessment of risk of bias across studies. Domain-level judgments and signaling question responses are presented according to ROBIS guidance.

| Domain | Signaling Question | Judgment | Rationale |
| --- | --- | --- | --- |
| Domain 1: Study eligibility criteria | 1.1 Did the review adhere to pre-defined objectives and eligibility criteria? | Y | Eligibility criteria and objectives were predefined in the protocol and Methods section. |
|  | 1.2 Were the eligibility criteria appropriate for the review question? | Y | Criteria appropriately addressed sex-specific biomechanical differences in PFP. |
|  | 1.3 Were eligibility criteria unambiguous? | Y | Inclusion and exclusion criteria were clearly reported. |
|  | 1.4 Were restrictions based on study characteristics appropriate? | PY | Restriction to sex-stratified biomechanical studies limited eligible studies but was appropriate for the review question. |
|  | 1.5 Were restrictions based on information sources appropriate? | Y | No language or publication date restrictions were applied. |
|  | Overall concern for Domain 1 | LOW | Eligibility criteria were clearly defined and appropriate. |
|  |  |  |  |
| Domain 2: Identification and selection of studies | 2.1 Did the search include an appropriate range of databases/electronic sources? | Y | Multiple databases and supplementary searches were performed. |
|  | 2.2 Were additional methods used to identify relevant reports? | Y | Hand-searching and ClinicalTrials.gov screening were conducted. |
|  | 2.3 Were search terms and strategy likely to retrieve eligible studies? | PY | Search strategy was comprehensive, although sex-stratified biomechanical studies are inherently limited. |
|  | 2.4 Were restrictions based on date, format, or language appropriate? | Y | No inappropriate restrictions were applied. |
|  | 2.5 Were efforts made to minimize errors in study selection? | Y | Screening was independently performed by two reviewers. |
|  | Overall concern for Domain 2 | LOW | Comprehensive and reproducible search strategy. |
|  |  |  |  |
| Domain 3: Data collection and study appraisal | 3.1 Were efforts made to minimize errors in data collection? | Y | Data extraction was independently performed by two reviewers. |
|  | 3.2 Were sufficient study characteristics available? | Y | Detailed participant and methodological characteristics were reported. |
|  | 3.3 Were all relevant study results collected? | PY | Some outcomes could not be pooled because of heterogeneous reporting. |
|  | 3.4 Was risk of bias formally assessed using appropriate criteria? | Y | Modified Downs and Black checklist was used. |
|  | 3.5 Were efforts made to minimize errors in risk of bias assessment? | Y | Independent assessment with consensus resolution was performed. |
|  | Overall concern for Domain 3 | LOW | Appropriate and standardized appraisal methods were used. |
|  |  |  |  |
| Domain 4: Synthesis and findings | 4.1 Did the synthesis include all relevant studies? | Y | All eligible studies were included in the qualitative synthesis. |
|  | 4.2 Were all predefined analyses reported or departures explained? | PY | Meta-analysis was limited by methodological heterogeneity and study availability. |
|  | 4.3 Was the synthesis appropriate? | Y | Quantitative synthesis was restricted to methodologically similar studies. |
|  | 4.4 Was heterogeneity addressed appropriately? | Y | Heterogeneity was formally assessed using I² and tau² statistics. |
|  | 4.5 Were findings robust? | PN | Funnel plots and sensitivity analyses were limited by the small number of studies. |
|  | 4.6 Were biases in primary studies addressed in the synthesis? | PY | Study quality was considered during interpretation of findings. |
|  | Overall concern for Domain 4 | UNCLEAR | Limited number of studies restricted publication bias assessment and robustness analyses. |
|  |  |  |  |
| Overall ROBIS judgment | Risk of bias in the review | LOW | Despite limitations related to the small number of included studies, the review methodology was comprehensive and transparent. |

Y = yes; PY = probably yes; PN = probably no; N = no; NI = no information.
